# A Treatise on Verminous Diseases, Preceded by the Natural History of Intestinal Worms, &c. &c.

**Published:** 1821-03-01

**Authors:** 


### Medico-Chirurgical Review,

AND

### JOURNAL OF MEDICAL SCIENCE.

(Analytical Series.)

Nec tibi quid liceat sed quid fecisse decebit
Occurrat mentemque domat respectus honesti. CLAUD.

VOL. I.] MARCH 1, 1821. [No. 4.

### I.

*A Treatise on Verminous Diseases, preceded by the Natural History of Intestinal Worms and their Origin in the Human Body.* By VALERIAN LEWIS BRERA, Professor of Clinical Medicine in the University of Pavia. Translated from the French, with additions, by J. G. COFFIN, M. D. of Boston, United States. One volume, octavo, pp. 363, with five plates.

IT was, and perhaps is still, the opinion of some philosophers, that this earth—this " huge globe we tread on," is but an enormous animal, and man, with all living creatures, but parasites that feed on their common parent. However this may be, it is at least certain, as Dr. Gregory has elegantly expressed it, that—" ipse animalium dominus hujusmodi animantibus predæ est et domicilio, quæ vel extrinsecus invadunt, vel intus habitant, et viscera adhuc vivi rodunt, sæpe cum insigni malo, et vitæ discrimine, nec raro ipsius jactura." It is somewhat remarkable that, in these days, when every the most trivial disease has one or more monographers, the important subject of WORMS should have led to no *ex professo* treatise (as far as we know) in our own language. Dr. Coffin has ably supplied this defect, by giving to his countrymen a translation of Professor Brera's excellent work; and all we can do, for such of our brethren here as cannot import either the original or translation, is to lay before them as comprehensive an analysis of Dr. Coffin's translation as our limits will permit, and as concentrated a record as literary labour can accomplish.

The able Italian Professor makes but one class of human intestinal worms, which he divides into five species, viz. the

Tænia, Vermis Vesicularis, Tricocephalus, Ascaris Vermicularis, and the Lumbricoides.

1. *Tænia.* This is a very long worm, apparently formed by a chain of flat articulations, united together by a border varying in breadth and thickness. The peculiarities of the joints, and of the perforated papillæ on the borders of some tæniæ, are owing, Dr. B. thinks, to difference of age, and abundance or deficiency of food. In mammiferous animals the length of the tænia is generally from nine to twelve feet; but in man, from twenty five to thirty yards. Some have been voided nearly 200 yards in length!

The head is sometimes so small that it cannot be distinguished without a microscope. It resembles a small tubercle which rises on the anterior extremity of the body, called neck. It is furnished with four apertures, which are eminences in some, depressions in other, worms. From each of these apertures proceeds a canal for nourishment, extending to the various articulations. On the heads of some tæniæ are recognized by the microscope small hooks, arranged in the form of a double crown; in others, there is no such apparatus, but a kind of mouth. The joints of the tænia diminish in size as they recede from the middle to either extremity of the animal, the tail terminating in a piece quite truncated, and raised on its sides in form of two hooks rounded at their summit. We have no exact description of the interior organs of the worm. We only know that it is oviparous, because in every joint there is an ovary containing a prodigious quantity of eggs of different sizes. These ovaries expel their eggs through the perforated papillæ of the sides;—it is therefore reasonable to suppose that the animals are hermaphrodites. The tænia is very tenacious of life. They have been known to live twelve hours in boiling veal broth, and come out as brisk and active as they went in. The tænia generally occupies the small intestines; occasionally it is found in the stomach. The head is usually towards the upper, and the tail towards the lower, portion of the gut. It is said that the head is firmly fixed in the mucous membrane, whence it is with difficulty dislodged. No one can be freed from the tænia till the head is evacuated.

Professor Brera divides the tænia into two varieties or species; viz. the armed and the unarmed tænia. The first, the tænia cucurbitina, or solitary worm, is that most usually discharged from our patients, and is altogether peculiar to man. With the naked eye we see its head armed with two pointed and protuberant appendages, by analogy called crotchets or fangs, which constitute the specific character of this species.

"If the anterior part of the head of this tænia is examined with the microscope, stretching it a little, the fangs, which are seen by the naked eye, are extended into a small crown, perfectly circular and stellated, in the centre of which is the tube." 35.

Werner has demonstrated that the organs constituting the two sexes exist in each ring, and thus proved its hermaphroditism.

The unarmed human tænia has been termed the *tænia lata*. It is flat, like a ribbond, of a white colour, and usually of a coarse, dense, and membranous structure. In general the joints of the neck are very thin and delicate, being almost imperceptible. Those that follow approximate the figure of a square, gradually increasing in width, thus continuing to the tail; which resembles a truncated piece. The longest human tænia lata which Pallas ever saw, was from eighteen to twenty feet. The head is very minute, and like the other species, is furnished with four lateral papillæ, and a central papilla and tube. This central papilla has not the crown of fangs which encircles the tube of the armed tænia. This worm is very rare.

II. *Vermis Vesicularis.* Joseph Ricci of Pavia, about 55 years of age, had been three months subject to intermittent fever, and violent mental affections. On the 26th November 1797, he was suddenly seized with severe pain in the upper part of the head. He cried out for help, and instantly fell down in a fit of apoplexy, which terminated fatally the following day.

"On examining the body, and finding nothing remarkable in the external substance of the brain, we attempted to open the two lateral ventricles, and found them filled with a bloody serum. Here an unexpected phenomenon presented itself; two large clusters of hydatids extended along the branches of the plexus choroides to which they were intimately attached, so closely that to separate them I was obliged to tear the substance of the plexus. Each cluster of hydatids was about two inches in length, large and extended at its inferior extremity, which floated at the bottom of the ventricles; the summit terminated by a long chord, folded in various directions, and was strongly attached to the partition which separates anteriorly the two ventricles.

"This double collection of hydatids so regularly disposed, being removed from the brain and attentively examined, we saw that each little bladder contained a real worm, of a structure quite singular." *Brera, p.* 41.

These worms had heads similar to those of the tænia, joined to a vesicle full of water, and very curiously organized. Each vesicle seemed to be formed of three different membranes—

the external thin, transparent, glistening—in the middle coat was seen an arrangement of slender circular fibres, which extender over another villous membrane that lined the inner surface of the vesicle. The vesicles contained nothing but water, without any appearance of an internal organ for the natural functions. This worm has been found in the brain, and in various parts of the human body.

III. *Tricocephalus.* This is exceedingly rare, and need not detain us long. It is in the form of a spiral line, about half a line in diameter, the external surface presenting an assemblage of small transverse rings. Its length is from one to two inches.

IV. "The *Ascaris Vermicularis,* which has received divers names by authors, is a round, filiform worm, fine and slender at both ends, from four or five lines to an inch in length. The vivacity with which it moves, skips, and bounds, is singular. If touched with a finger, or brought near the flame of a candle, its body contracts some lines in a surprising manner. It is perhaps to this contractility that we are to attribute those enormous irritations of the intestines, and particularly of the anus, which torment the sick, especially children, who are most subject to them.

"The surface of this worm is full of wrinkles, which seem to be formed by a multitude of rings. Its anterior extremity is obtuse, its posterior end, or tail, is shiny and slender. 49.

The domicile of this worm is generally the cavernous cells of the colon and rectum. It is never found alone, but in conglobate masses of other worms of the same genus. The mucus of the intestines and vagina is that for which this worm has the greatest predilection.

V. The *Lumbricoides* is perfectly round, about the size of a writing quill, and commonly from six to ten fingers' breadth in length. Its colour is white, the whole surface of the body wrinkled and annular, tapering towards both extremities. In the head, examined with the naked eye, we see three beautiful hemispherical eminences which insensibly terminate in a very sharp point, in the centre of which is an aperture.

"In the living worm, on the contrary, we see that these three hemispherical protuberances are pyramidal, with a convex base truncated exteriorly with a very sharp piercing point, in such fashion as to be compared to the divisions or claws of common pincers. With these protuberances the lumbricoides attaches itself to the membrane of the intestines, and even penetrates it, and when sucking up the mucous secretions, it moves these eminences alternately like three jaws; thus the worm opens and shuts its triangular mouth, furnished with a tube which it can put out or draw in. A mechanism so well understood proves that these protuberances are tissues or textures of muscular fibres." 56.

Children are much disposed to this worm, and adults are not always free from it. They prevail most in persons poorly nourished, and full of viscous humours. They are generally found collected together in great numbers.

### VERMINOUS DISEASES.

We have passed over our author's third lecture on the origin of worms, as we are *defenders of the faith*, "omnia ex ovis," in opposition to the advocates of equivocal generation.

The morbid symptoms attending worms in the intestines will be proportionate to the number and size of these animals; to the sensibility of the parts they occupy, and the general morbid diathesis, whether as cause or effect. Children are most liable to the lumbricoides and ascarides—adults to tænia and vesicular worms. The symptoms of worms are undoubtedly often ambiguous; yet there are certain leading phenomena which may at least admonish the practitioner of the probability of the existence of these animals.

"In persons attacked by worms, the colour of the countenance is changed; it is sometimes red, then pale, or leaden-coloured; a half circle of azure appears under the eyes, they lose their vivacity, and are fixed and motionless with regard to surrounding objects; they are sad and dejected; the lower eyelids swell, and the pupils are evidently dilated. At other times the eyelids are yellowish, and the same tint extends over the white of the eye. There are also insupportable itchings in the nostrils, with occasional hæmorrhage from the same parts; headache is frequent, expecially after taking food; this is sometimes so violent as to produce delirium and phrenitis.

"The mouth is full of saliva, and exhales a fetid and verminous odour; there is grinding of the teeth; uneasy and agitated sleep, and great thirst. Sometimes somnambulism renders the patient timid. Fainting, vertigo, and tingling of the ears, augment the morbid state of the sufferer. The cough is dry and convulsive, sometimes stertorous and even suffocating, respiration is difficult, and sometimes attended with hiccough; speech is interrupted, and in some instances entirely suppressed. The mouth is frothy, and there is palpitation of the heart; the pulse is hard, frequent, rapid, and intermittent.

"The belly is tumid and troubled with borborygmi; there are eructations, nausea, reaching to vomit, and vomiting. At one time there is no appetite, at another it is so great that the patient is compelled to take more food than ordinary. The belly swells and is the seat of severe pains; there is a sense of pricking and tearing which is not fixed, but wanders over the whole abdominal cavity; these sufferings are aggravated when the stomach is empty, and immediately cease on taking food. The bowels are sometimes relaxed, sometimes costive.

"The urine is crude and turbid; the excrements fetid; cardialgia afflicts the patient and sometimes destroys him; the body is emaciated, though the patient eats much; and violent itching of the anus sometimes occasions fainting.

"At other times tenesmus aggravates the pains of these parts. Languor, anxiety, listnessness, and extravagance in conduct discourse and the intellectual functions, are observed in persons harrassed with worms." 143.

We are not to suppose that the union of all these symptoms is requisite to enable us to judge of the presence of worms; the principal of them are sufficient, especially dilatation of the pupil, abundance of saliva in the mouth, irregularity of appetite, emaciation, a pricking sensation of the stomach, tumefaction of the abdomen, anxiety, loathing of food. Professor Brera has seen pains of the joints, resembling arthritic rheumatism resulting from worms. In fact, the sympathetic affections produced by the irritation of worms in the primæ viæ, are innumerable, and exceedingly anomalous. Weikard relates the case of a woman who was long harrassed with headache, spasmodic affection of the eyes, vertigo, sub-apoplectic attacks, and occasionally temporary blindness. One day, feeling something in her nose, she extracted, with a hook, a living lumbricoides, and then three more. Some anthelmintic remedies now prescribed, brought away seven worms, *per anum*, when the woman was perfectly cured of her distressing complaints.

In another instance, a man had complained for three years of a fixed obtuse pain in the right hypochondriac region. He was afterwards attacked with a slow fever, and died much emaciated.

"On opening the body, the right lobe of the liver was found hard and large; introducing the scalpel, a great quantity of yellowish serum passed out, with several hundred hydatids of different bigness. There was every reason to believe they were social vesicular worms." 145.

Worms have been known to excite apoplexy in the brain, and nephritis, when situated in the bladder. *Producunt aliquando erectionem molestam penis.*

*Symptoms of Tænia.* Pain in the belly, with a turning motion and weight in the side—occasional prickings or bitings in the region of the stomach—swelling of the abdomen at intervals, with sense of coldness there—appetite enormous, while emaciation continues, with sense of increasing weakness—complexion livid—pupils unusually dilated—eyes suffused with tears—vertigo with nausea—vacillation of the legs, and sometimes convulsive tremblings of the whole body—occasional evacuation, *per anum*, of small substances resembling the seeds of the lemon or gourd, which are portions of the marginal papillæ of the worms.

As the head of the armed tænia is furnished with pointed fangs, it sometimes attaches itself with such force to the mucous membrane of the intestines, as to produce the most severe, and even deadly symptoms, since the membrane is mangled, and inflammation, or even gangrene, may be the consequence. "A singular symptom of this tænia is a frequent sense of tension or tightness in the nose."

*Tricocephalus*. These worms, although not possessing a biting organ, may yet irritate the intestines by their presence; while, collected in great numbers, they deprive the system of its requisite nourishment, and thus diminish the strength.

*Ascaris Vermicularis*. These generally reside in the large intestines, vagina, and other mucous parts. They excite in the large intestines, particularly in the rectum, a dull feeling of irritation, or a tedious and unsupportable itching, or sometimes very acute and cutting pains.

"They are united into conglobate masses with other worms of the same family; the inner surface of the intestines is entirely altered by the irritation produced by thousands of these worms, and the want of mucus, which their eating this fluid occasions, renders these parts more sensible and irritable." 152.

Infants and enfeebled persons having more mucous substances in their bowels, and being also more irritable than adults and robust constitutions, these worms prevail more in the former, than in the latter, and produce in these individuals, more serious inconvenience.

"Those portions of the intestinal canal which are supplied with nerves from the branches of the intercostal nerve being irritated by worms, the effects which hence result and which have often been observed, are convulsive cough, grinding of the teeth, itching of the nose, and various other verminous affections, from sympathy.

*Lumbricoides*. These being possessed of a cutting, sharp point, they often cause pungent and rending pains, especially about the umbilical region. Colic and borborygmi are symptoms peculiar to this kind of worm, which sometimes makes its way through the walls of the intestines. It is fortunate for the human machine that this animal possesses great sensibility.

"Air, and cold water, throw them into a state of asphyxia, and the peristaltic motion of the intestines when quickened, or the use of a drastic purge, is often sufficient to expel them from the body. For this reason, when these worms have once descended into the large intestines, they are easily evacuated." 154.

### SYMPATHETIC AFFECTIONS FROM WORMS.

The doctrine of sympathy between the digestive organs and almost all other parts of the body, was known to Hippocrates, who observes—"in corpore humano conflexus unus, conspiratio, una et omnia consentientia." *De Alimentis.* In all anomalous and rare diseases therefore, the intelligent physician will enquire whether his patient has, or has had, any symptoms of worms, since the irritation produced by these animals in the stomach or intestines may derange the whole animal economy, and prove the cause of the most violent morbid affections, even in parts of the body most remote from the abdomen, and particularly the skin. Hence arise, in those troubled with worms, intermissions of the pulse, palpitations of the heart, syncope, vertigo, loss of speech, blindness, buzzing in the ears, mental dejection, restless sleep, hiccup, convulsions, epilepsy, and many other diseases. Here our author gives it as his opinion, that what have been denominated "worm fevers" were really low fevers, "during which worms multiply and grow in those parts of the body that are most enfeebled."

*Treatment.* Our able author observes that the great or fundamental principle in the treatment of worms is to prevent their generation—that is to strengthen the system, so that they may not only be dislodged but rendered incapable of reproduction.

"In the treatment of verminous complaints in general, such remedies as strengthen the body, at the same time that they diminish the morbid secretion of mucus, and resist the decay and consumption of all the parts, give action to the organs destined to the natural functions, annoy the worms, destroy them, and excite throughout the system that energy which is so necessary to expel them, and to prevent their further increase; the remedies that produce all these effects, accomplish the necessary indications." 189.

Professor Brera properly remarks that it is of little use to forcibly expel worms by drastic purgatives, and leave the predisposition to their subsequent increase unsubdued. He is very sceptical respecting the *specific anthelmintic power* of medicines. He is more inclined to account for their efficacy on general and well-known principles. Drastic purgatives, though they may be proper in robust constitutions, generally occasion serious mischief when given to enfeebled patients. "In these cases we should effect the desired end, by employing remedies which excite and strengthen the animal frame, without altering the natural secretions of the fluids." Here our author enters upon a *catalogue raisonnée* of the most approved anthelmintics generally speaking, after which he enumerates those medicines which experience has proved to be powerful in expelling particular species of worms.

We shall endeavour to follow our author, passing entirely over some of the remedies, and abridging the observations in all cases.

### VEGETABLE VERMIFUGES.

1. *Allium Sativum, Garlic*, has been proved by Rosentein and Tissot to be capable of expelling worms, especially the tænia.

2. *Artemisia Santonica*, or worm seed, is well known to have considerable power over the lumbricoides particularly. The dose is from two grains to a drachm.

3. *Jalap*, probably from its disagreeable smell and nauseous taste, is a useful auxiliary, at least, to other anthelmintics.

4. *Assafœtida* has been often usefully employed in several diseases resulting from worms, particularly in the spasmodic class. It is sometimes combined with other medicines, as myrrh, the black oxide of iron, calomel, &c. Assafœtida enemata are useful in the ascarides.

5. *Oil*, as closing all the speracula of worms is very inimical to these animals. The oil of walnuts has been particularly extolled.

6. *Camphor*. Pringle long ago demonstrated the anthelmintic powers of camphor. The celebrated Moschati generally prefers it to other vermifuges. Half a drachm is dissolved in a pint of water, to which a drachm of gum arabic is added, and this mixture is given in spoonfuls. Or injections of stronger solutions than the above are thrown up.

"The employment of camphor is also attended with this precious advantage, that it counteracts the predisposition to the further development of verminous seeds.

"I have always used it with the greatest success; and I cannot too strongly recommend its use to physicians in worm complaints, whether given in the mode already mentioned, or some other, or combined with other remedies." 199.

7. *Tanacetum Vulgare*, the common tansy, has been long celebrated; but needs only to be enumerated here on the list of anthelmintics.

8. "Aloes, rhubarb, the gratiola officinalis, gamboge, chamomile, and particularly sulphureted scammony, *(diagrède sulphuré,)* and other similar articles, are also remedies commonly used for the expulsion of worms. I have not spoken of these substances singly, because these drastics being usually combined with vermifuge remedies, vegetable or mineral, cannot in strict reasoning be directly classed with those medicines, which we use to expel worms from the body, and to prevent the development of verminous seeds." 203.

### MINERAL VERMIFUGES.

9. *Murias Ammoniæ*, combined with rhubarb or jalap, is considered by Block as a very efficacious vermifuge.

10. *Iron*, in consequence perhaps of its tonic powers, is a well-ascertained anthelmintic, tending both to destroy worms, and prevent their subsequent generation. The sulphate of iron is considered to be the best preparation, as possessing the greatest astringent force, and being powerful in moderating excessive secretion of mucus in the bowels. To children Professor Brera gives it in doses of from two to ten grains; and to adults in doses of half a drachm to a drachm. These are large doses. Our author thinks that its virtues are much enhanced by conjunction with cinchona, valerian, jalap, assafœtida, &c.

11. *Mercury.* Quicksilver can have no specific effect against worms, since those who work in the mercury mines of Almada, in Spain, are peculiarly subject to worms, though these people absorb such enormous quantities of that mineral that small globules of mercury are evacuated with the stools. The same thing happens in the mines of Lydria, and in the laboratories of Chemnitz, in Hungary. Besides this, Rosenstein has administered mercury in several cases, even to salivation, without being able to expel a single worm.

> "Mercury, given in the state of oxide, acts on the solids as a powerful stimulant, since by its use, the pulse acquires great force, and the secretions and excretions are augmented. In this way several of the oxides of mercury have been very efficient in expelling worms, and in curing verminous affections. Among these the submuriate of mercury is to be preferred." 213.

Ptyalism should never be produced in these cases.

12. *Petroleum* is famous at Montpellier against worms. Rosenstein, many years ago, relates the case of a man who was delivered of a tænia by a dose of petroleum and oil of turpentine.

13. *Stannum* was considered vermifuge ever since the days of Paracelsus. Of zinc, sulphur, and some other minor anthelmintics, we shall not here take notice.

### TREATMENT OF TÆNIA.

Our author justly observes that the same treatment which succeeds in a robust, will often fail in a weak, or cachectic constitution. Drastic purgatives may expel the tænia from the one, while tonics aud stimulants may be the best remedies in the other.

1. *Rosenstein* expelled tæniæ by first giving a purgative, and after its operation had commenced, causing the patient to drink repeated draughts of *cold water*, which, by depriving the worm of the power of moving the neck and fixing the head into the folds of the intestines, rendered it liable to be detruded by the peristaltic action of the intestines. Darilias, Lindhult, and some others followed this plan.

2. " *Chabert.* The essential oil of turpentine, combined with petroleum, has already been noticed, as well suited to expel tæniæ. The remedy of *Chabert* consists in the distilled oil of turpentine with the liquid carbonate of ammonia; this mixture he assures us is a very powerful and infallible means of expelling the tænia from domestic animals. Repeated observations prove, that though this remedy acts with activity and energy against tæniæ, it produces not the least disorder in the system. It is also to be desired that it should be adopted by physicians to expel tæniæ from the human body, since we have seen that the essential oil of turpentine, muriate of ammonia, and carbonate of liquid ammonia, are also remedies, which have been advantageously employed both against tæniæ and lumbricoides." 225.

Chabert published on this subject in the year 1781, and our author's lectures were delivered in the years 1798 and 1799, consequently all claim to priority or originality is taken away from the practitioners who first introduced oil of turpentine as a remedy for tape-worm in this country. The earliest mention which we have seen made of the medicine is by Dr. Bateman in the Edinburgh Journal for April 1810, and is as follows:—

" I may here mention, that in consequence of a communication from Dr. Southey, of Durham, to my colleague, Dr. Laird, in which it was stated that the *oil of turpentine* had been discovered by Dr. Fenwick to have been used, with considerable success, in the expulsion of *tape-worm*, by a mechanic of that city, this substance has been administered in doses of from *half an ounce* to *two ounces*, by several physicians of public charities in London, and it has appeared to be an active antidote to that troublesome animal, in a great majority of instances. In a considerable number of cases its exhibition has been followed, in the course of a few hours, by the discharge of a *dead* tænia, of many yards in length. The oil of turpentine may be swallowed with scarcely more inconvenience than so much gin, and its effects are nearly the same when taken in large doses, viz. some degree of vertigo, or an approach to intoxication. It generally acts as a speedy purgative, and produces no unpleasant effects on the bladder: but, in a very few instances, it has occasioned a distressing sense of heat in the stomach, with considerable sickness; and, in one or two cases, has produced strangury. Even in these few cases, in which it has failed to expel the tænia, it has commonly afforded great relief to the painful feelings which were believed to originate in the presence of the worm."* 253.

In the 7th volume of the same Journal, Dr. Bateman again mentions two cases of tape worm, in both of which turpentine, to the amount of ℥iss, was given—"and in both it was followed by a discharge of a very considerable length of the worm."

In the 9th volume, Dr. Bateman states the case of a patient affected with tape-worm, where the oil of turpentine afforded certain, but temporary relief from this troublesome parasite. The patient took the medicine five or six successive times, and invariably with an expulsion of a considerable length of worm, to the extent, upon the whole, of many yards. But always in two or three months his pains returned, and he was forced to repair to the dispensary for a repetition of his dose.

After this the periodical journals exhibit numerous cases of the successful exhibition of oil of turpentine for tænia. Mr. Hartle, of Antigua, has published some interesting cases, and made some judicious observations on the subject. He says, "I am fully persuaded, that a large dose is less likely to affect the urinary organs, than a small one; a large dose acts immediately as a cathartic, and purges itself off before the absorbents have time to play on it." Mr. Hartle thinks that the effects of tænia are often overlooked, in the West Indies, or confounded with chronic dysentery.

"The patients are generally, at least those that I have seen, meagre and emaciated, with a voracious canine appetite, never satisfied with any quantity of food, and wearing all the appearance of having been starved. Griping pains of the belly, particularly in the mornings before breakfast, with one or two watery stools, a grumbling noise of wind in the intestines, succeeded by pain, pains in the stomach, and occasionally passing pieces of the worm. I would recommend to every medical gentleman to desire his patients to observe particularly their stools, by which means he will often find out the true disease, and be relieved from that anxiety which must naturally

* In the second volume of the Medico-Chirurgical Transactions, for the year 1811, Dr. Fenwick's paper is published. It was read at the society on the 2d of January, 1810, and the allusion to it by Dr. Bateman is in April 1810. Dr. B. of course, heard the paper read in January.

be excited in the mind of every medical man, when he finds that his remedies have failed to give relief." *Ed. Journal, vol.* 14, *p.* 482.

Dr. Coffin, the American translator of Brera's work, informs us that ol. terebinth, is now generally used in the United States, and he thinks "it will be found equally effectual in killing all the species of this worm and the lumbricoides." The greatest inconvenience Dr. Coffin experienced from the exhibition of this medicine is the thirst which follows, and the prohibition of taking fluids for many hours, which is generally enjoined. To obviate this, he recommends the stomach and bowels to be well evacuated before the spirit of turpentine is given; if the latter be in a sufficiently large dose, it will commonly pass through the bowels in an hour and a half, or even less. As soon as any turpentine begins to pass *per anum*, whether the worms appear or not, the patient may be allowed to drink freely of any bland fluid.

Upon the whole, there can be little doubt but that oil of turpentine is the best remedy hitherto discovered for the expulsion of tænia. But as different constitutions will occasionally require different means, it is proper in an eclectic article of this kind to enumerate the principal methods which have been employed by different practitioners.

3. *Method of Nouffer.* Towards the middle of the last century, Madame Nouffer's remedy against tænia excited considerable attention. The medicine was three drachms of the root of the male fern, in powder, mixed with four or six ounces of the distilled water of male fern, or lime-tree flowers, taken in the morning. The dose, of course, was graduated for children. Two hours after taking the powder, the patient is to swallow the following bolus:—Take of calomel, and dry resin of Aleppo scammony, of each twelve grains; of gamboge five grains; form into a bolus with hyacinth confection. This, by the way, was a tolerably good vermifuge of itself, and doubtless contributed more towards Mad. Nouffer's success than the fern powder.

4. *Method of Odier.* The remedy of this celebrated physician was no other than castor oil. It both kills the worms and expels them. Adults should take three ounces of the oil, and children a tea-spoonful several times a day. Professor Brera considers that this remedy is chiefly applicable to cases of unarmed tænia. "I can, however, say, that it sometimes serves wonderfully well to expel also the armed tænia." Lelle advises that the oil be taken at bed-time, and ten grains of gamboge the next morning.

5. *Method of Desault.* This celebrated physician "proposed an ingenious and bold expedient, that of administering alternately a mercurial friction, and a purgative of calomel in a large dose."

6. *Method of Alston.* This is by tin. But, as Professor Brera properly observes, this method is objectionable on account of the danger of lead or arsenic being mixed with the tin. He himself saw the colica pictonum, and paralysis of the lower extremities produced by taking the filings of tin.

*Method of Mathieu.* His method, which begins to prevail, consists in the administration of two electuaries, compounded of the filings of English tin, the powdered root of male fern, semen-contra, scammonia of Aleppo, gamboge, and sulphate of potass.

"The electuaries which M. Mathieu administers to his patients are mild; the first is marked A. the second B.

"*The First Electuary*, A. Take an ounce of very fine English tin filings, six drachms of the root of the polipodium filix mas, half an ounce of semen santonicum, a drachm of the resinous root of jalap, and of sulphate of potass, and of honey sufficient to make an electuary.

"*Second Electuary*, B. Take two scruples of the pulverized resinous root of jalap, and of sulphate of potass, one scruple of scammony from Aleppo, ten grains of gamboge, and of honey sufficient to form an electuary.

"Those who may be inclined to adopt this method to expel tæniæ, must observe the four following rules:—

"1. For some days previous, the patient is to be confined to a suitable diet, that is, he is to eat salted substances,—for example, herrings, light porridges and broths, and leguminous articles.

"2. The treatment is begun by administering to the patient, every two hours, a tea-spoonful of the electuary A. This course to be continued two or three days, till the worm is perceived to be in the intestines, and then,

"3. The patient is to take electuary B, and of this he also takes, every two hours, a tea-spoonful, till the worm is expelled.

"The discharge of the worm is facilitated by taking some spoonfuls of fresh oleum ricini, or by some clysters of the same oil.

"4. The age, sex, and temperament of the patient may require a considerable modification of the dose of these remedies; for this reason the treatment ought to be directed and modified by a well informed physician.

"Finally, it is to be borne in mind that the virtue of the electuary A. depends in great part on the root of the polypodium filix mas; hence this root should be fresh, and its internal hard part only should be reduced to powder. 345-6-7.

The French translators here state that they saw Dr. Bourdier, of Paris, administer the following remedy, with great success in both species of tænia.

"Pour a drachm of sulphuric ether into a glass of the decoction of male fern, which the patient is to take fasting; four or five minutes after, an injection of this same decoction, with two drachms of ether, is to be thrown up. One hour after, give two ounces of oleum ricini, and one ounce of the syrup of peach blossoms. This treatment is to be continued for three days. The worm is commonly discharged but half organized.

"When the worm is in the stomach, success is certain; when in the intestines, the treatment, after some time, is repeated; then Dr. Bourdier prescribes an enema of decoction of fern and two drachms of sulphuric ether, immediately after the patient has swallowed the etherated potion." 241.

8. *Arsenic.* Dr. Coffin has known Fowler's solution destroy the tænia in several cases.‡ Dr. Fisher, of Massachusetts, also observes, that the tænia may be destroyed by Fowler's solution. For this purpose the patient should take it, two or three times a day, in as large doses as the stomach will bear: and continue the use of it till the worms are destroyed. Hitherto this remedy has not disappointed me in a single instance."

In this country Dr. Girdlestone, of Yarmouth, states in the 15th volume of the Medical and Physical Journal, that he has, for some time, prescribed the solutio mineralis in cases of tape-worm. "This medicine," he says, "with the use of purgatives, brings away larger portions than any purgative medicine without it. And I have found the solutio mineralis a most powerful destroyer of the ascaris lumbricoides."

9. *Pomegranate.* Dr. Pollock, of Bengal, has stated that a number of cases of tænia were cured by decoction of pomegranate. "In some of these the tænia had acquired an enormous length, and in some of them it was received in tepid water, and lived for several hours after it was passed." *Ed. Journal, vol.* x. *p.* 419.

*Treatment of the Ascaris Vermicularis.*—"Clysters of *geoffroya surinamensis*, of *assafœtida*, of *veratrum sabadilla*, of tepid milk well salted, or of simple water salted, are the best remedies to drive these worms from the large intestines. Enemas of oleum ricini, and plugs of soap smeared with this oil, are very useful. Tenesmus, hæmorrhoids, swelling, tension and inflammation of the anus, symptoms sometimes occasioned by ascarides vermiculares, particularly when there is inflammation of the intestines, ought to be treated with clysters and emollient fomentations, and, in general, agreeably to the indications of peculiar circumstances." 292.

We may add, that injections of lime water here proved very serviceable in this species of worm. We must endeavour to supply the loss of the natural mucus destined to lubricate the inner surface of the intestines with enemata of gelatinous substances. Ascarides, though feeble worms, are with difficulty destroyed. The minute embrions of the female ascaris, though deposited alive, are not visible at the time, and therefore, patients who too suddenly abandon the curative regimen, are again attacked when they think themselves well. The use of injections alone is not generally sufficient to destroy these worms, which sometimes ascend the alimentary tube as far as the small intestines, or even the stomach. Mr. Charles M. Clarke says:—

"A strong decoction of the semen santonici is the most efficacious of all the injections in use. With this the rectum should be filled; but the quantity thrown up should never be so great as to produce great distention of its cavity, lest the coats of the bowel being stimulated, it should contract hastily and expel the glyster, which acts with more certainty, if it remains for some time. This operation, repeated for a few successive days, will seldom fail to remove, for a time, the ascarides and the symptoms they produce.

"Purgatives employed alone are of little service; but during the use of the glysters they ought to be occasionally exhibited." 294.

In addition to injectious, we have generally ordered small doses of calomel for two or three nights, and then a cathartic of rhubarb and jalap, or scammony, on the third morning. In a majority of cases, this treatment, with glysters of lime-water, oil, or assafœtida-solution will succeed. Where these fail, we think the spirits of turpentine should be tried.

*Lumbricoides.* Of all the remedies for lumbriciodes, Professor Brera thinks there is none equal to camphor.

"This substance, administered according to rule, expels lumbricoides with facility and promptitude, and at the same time strengthens the intestinal tube and the whole body, as we have already said. This remedy kills these worms in an instant, perhaps because its penetrating and volatile odour acts by restoring, in a surprising manner, the excitement of the first passages, and those parts, which are sympathetically connected with these organs, relieving the convulsions and spasms occasioned by worms, and preventing the cause of them." 299.

Perhaps, however, the dolichos pruriens, or cow-itch, may be ranked among our most powerful vermifuges, destroying not only the tænia, but also the lumbricoides and ascarides. The ripe pods are to be dipped in syrup, which is again scraped off with a knife. When the syrup is rendered as thick as honey by the hairs, it is fit for use. It may be safely taken from a tea to a table-spoonful in the morning fasting. Mr. Chamberlaine, who published on this subject, prefers common treacle to any other vehicle. In Massachusetts,

Dr. Coffin informs us, it is much used, and is more certainly and speedily beneficial, when it is preceded by an emetic or cathartic, or both; and when a purgative, as castor oil, is occasionally interposed. This should be done every second or third day, during the exhibition of this article, if there is the least costiveness.

In respect to hygiene, after worms are expelled, it is only necessary to state, that all those means which strengthen the system generally, and the tone of the digestive organs in particular, will be the proper preservatives against a regeneration of these disagreeable parasites.

We have now presented the prominent features of our learned author's work, and that of his able and intelligent translator. We have concentrated into a small space, a great mass of useful information, which we hope may prove acceptable to the profession, especially to the junior branches, as a concise reference on the subject of worms and verminous diseases.

